# Muscle Fatigue When Riding a Motorcycle: A Case Study

**DOI:** 10.3390/ijerph18157738

**Published:** 2021-07-21

**Authors:** Priscila Torrado, Michel Marina, Stéphane Baudry, Martín Ríos

**Affiliations:** 1 Research Group in Physical Activity and Health (GRAFiS), Institut Nacional d’Educació Física de Catalunya (INEFC), Universitat de Barcelona (UB), 08038 Barcelona, Spain; priscilatorradopineda@gmail.com; 2 School of Health Sciences, TecnoCampus Mataró, Universitat Pompeu Fabra, 08302 Mataró, Spain; 3 Laboratory of Applied Biology and Neurophysiology, Université Libre de Bruxelles, 1070 Bruxelles, Belgium; stephane.baudry@ulb.be; 4 Facultad de Biologia, University of Barcelona, 08028 Barcelona, Spain; mrios@ub.edu

**Keywords:** electromyography, motorcycles, muscle strength, forearm, hand strength, neurophysiology

## Abstract

This case study was conducted to assess muscle pattern, as measured by surface electromyography (sEMG), and its changes during a controlled superbike closed-road track training session. The sEMG signals were recorded unilaterally from biceps brachii (BB), triceps brachii (TB), anterior and posterior part of the deltoid (DA and DP respectively), flexor digitorum superficialis (FS), extensor carpi radialis (CR), extensor digitorum communis (ED) and pectoralis major (PM) during three rounds of 30 min. sEMG signals selected for analysis came from the beginning of the braking action to the way-out of the curves of interest. Considering the laps and rounds as a whole and focusing on the forearm muscles, ED was more systematically (84%) assigned to a state of fatigue than FS (44%) and CR (39%). On the opposite, the TB and DP muscles showed a predominant state of force increase (72%). Whereas the BB showed alternatively a state of fatigue or force increase depending on the side of the curve, when taking into account only the sharpest curves, it showed a predominant state of force increase. In conclusion, the fact that forearm muscles must endure a long-lasting maintenance of considerable activity levels explains why they easily got into a state of fatigue. Moreover, TB and DA are particularly relevant when cornering.

## 1. Introduction

Motorcycle road races last from 30 to 45 min, representing about 20 to 25 laps consisting of 12 to 20 curves. This profile requires thereby 200 brakes and 400 leans per race at velocities generally greater than 200 km/h [1] that should be managed with accurate synergistic muscle contractions from different part of the body, despite the development of muscle fatigue [2]. However, only a few studies have investigated muscle fatigability via surface electromyography (sEMG) in riders that were performed either in a laboratory environment [3,4,5] or outside the track [2]. At present, only two studies have reported an accurate fatigue assessment yielded during a real piloting setup [6,7]. Nevertheless, both studies monitored a pilot driving a motorcycle in a motorway or normal road environment, much less demanding and stressful than a racetrack.

Another limitation in studying muscle fatigability during track motorcycle race is related to the interpretation of changes in sEMG during force-varying contractions. It is widely accepted that muscle fatigability represents a progressive decrease in the capacity of an individual to produce high levels of force or to maintain steady force output, a decrease that starts from the beginning of the exercise [8,9,10]. However, such assessments are rather difficult in an “on-track” experimental set-up.

Another common technique to evaluate muscle fatigability is the surface electromyogram (sEMG), which records the electrical activity associated with muscle contraction. During sustained isometric submaximal contractions, fatigue cause an increase in sEMG amplitude (time domain analysis), and a decrease in the power spectrum (frequency domain analysis) [11,12,13]. sEMG amplitude increases could be explained by a combination of an enhanced recruitment of fibers with higher action potential [12] and an increased synchronization of the motor units [14]. On the other hand, power spectrum decreases could account for an indirect measure of the metabolic status of the muscle cell membrane [15], based on matched behavior with conduction velocity of the action potentials that propagate along the muscle fiber membrane, and muscle lactic acid, due to a restricted blood flow [16]. However, these electrical indices have some limitations during force-varying contractions [17]. Accordingly, Luttmann et al. [18] developed the joint analysis of sEMG spectrum and amplitude (JASA), which combines the time and frequency domains of the sEMG signal, allowing to define four quadrants [18,19,20,21]: (1) force increase (root mean square (RMS) and mean frequency (MF) increase), (2) fatigue (RMS increase and MF decrease), (3) recovery (RMS decrease and MF increase), and (4) force decrease (RMS and MF decrease). This approach allows to determine a reliable pattern of sEMG during repeated tasks with similar force production and has been successfully used to assess neuromuscular fatigue in occupational labor [20], such as a surgery [18], wheelchair maneuvers [19], virtual environments [22], construction [21], or cycling [23].

Very little information is available about the required muscular load during the different actions that take place during a motorcycle road race. In consequence, we could say that up to now, physical training programs in this sport have been based on empirical knowledge and not on scientific evidence. Therefore, the objectives of this study were (1) to assess the muscle activity changes that occur during riding on a road-race track, and (2) to find out whether muscle fatigue develops when riding a motorcycle during consecutive rounds of a training session on a circuit. We hypothesized that the most demanded muscles should be the flexor superficialis digitorum (FS), as the agonist of the brake-pulling action against the lever [4,24], accompanied by the extensor digitorum (ED) considered as the antagonist pair of the FS. Co-contraction of ED and carpi radialis (CR) is supposed to occur during the braking phase and entry of the curve because of their wrist stabilization role already observed in power grip tasks [25,26]. Based on the previous hypothesis, we supposed that at the end of the training sessions, at least some of these muscles should get into a fatigue state. Knowing the high inertial forces that must be managed by the motorcycle riders [1,27], we additionally hypothesized that a complex interplay should exist between the agonist/antagonist pair mainly responsible for the stabilization of the elbow (biceps brachii versus triceps brachii; BB/TB) as well as for the role of the shoulders, when transmitting forces from de handlebar to the rest of the body and vice versa. With respect to the last hypothesis, the occurrence of fatigue should change the leadership figures and synergies among these muscles.

The relevance of these data should be considered with respect to the difficulty to obtain reliable sEMG recordings while riders drove at high speeds carrying on all measurement instruments despite heat, sudation, and movement artifacts. This challenging experiment opens new area for applied research in motorcycling.

## 2. Materials and Methods

### 2.1. Experimental Approach to the Problem

**Figure 1:**
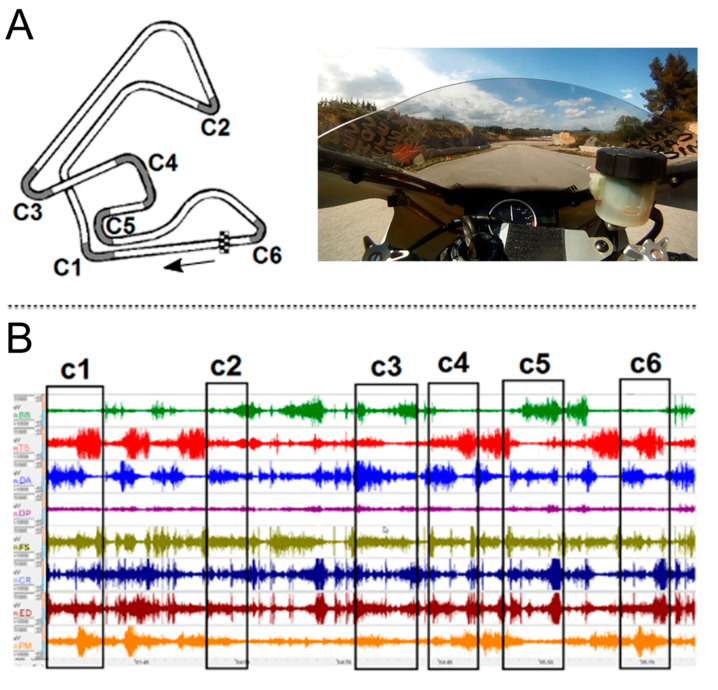
Section (**A**) shows the track layout and the most relevant curves. The layout of interest for each curve (beginning, apex and way out) is shaded in grey. Section (**B**) illustrates the registered sEMG signal of one lap and the analyzed sEMG sections corresponding to each curve.

The participant performed the tests riding a Yamaha R1 1000 cc (Yamaha Motor Company, Iwata, Shizuoka, Japan) prepared for racing in a closed-road track. In order to link the rider’s activity with the sEMG recording, we installed a video camera on the body fairing of the motorcycle to record the track-view and identify each curve sectors of the track (Figure 1A).

### 2.2. Participant

A healthy motorcycle rider, who participated at national competition level, volunteered in this study. He was free from known neurological or musculoskeletal disorders, such as the forearm chronic exertional compartment syndrome, so widespread among the population of motorcycle riders [28,29]. The age, body mass, and height of the participant were 48 years, 75 kg, and 177 cm, respectively. The criterion for inclusion in the study was related to the experience riding a large motorcycle engine (1000 cc, more than 170 CV) in road racing situations, as well as a consolidated knowledge of the racetrack layout. Prior to the data acquisition, written consent was obtained after informing the participant about the risks and rights of the study. The study was approved by the ethics committee of clinical research of the local sport administration (reference number 15/2018/CEICEGC).

### 2.3. Circuit

The circuit (Parcmotor Castellolí, Catalonia, Spain) had a length of 4140 m with a layout composed of seven right and four left curves of varying radii (from 100 m to 10 m) (Figure 1A). All curves had cones to signal their beginning, apex, and way out to better detect the corresponding sEMG signal. Only the sections corresponding to the most physically demanding curves where considered (Figure 1B). By demanding curves, we mean those where the velocities preceding the curve were always over 200 km/h and which required very aggressive braking followed by the greatest leaning angles of the motorcycle. That is, those sections of the layout that solicit, from riders, high intensity braking actions combined with technical maneuvers, which overall expose them to substantial physical stress [1]. The criteria chosen to select these track sections came from a sum of biomechanical factors that generate the highest inertial forces, only manageable with a substantial contribution of muscular work [27]. The track was dry and the average outside temperature during the session was 18 °C, with a relative humidity of 68%.

### 2.4. Surface sEMG Recording

An ME6000 electromyography system (Mega Electronics, Kuopio, Finland) was used to register the sEMG signals of the biceps brachii (BB), triceps brachii (TB), anterior and posterior part of the deltoid (DA and DP respectively), flexor digitorum superficialis (FS), extensor carpi radialis (CR), extensor digitorum communis (ED), and pectoralis major (PM). Muscles from the right side of the body were chosen as both the braking and gas modulation gestures are performed with the right hand. After shaving, abrading, and cleaning the skin with alcohol-soaked paper, surface electrodes (Ambu Blue Sensor, M-00-S, Ballerup, Denmark), were placed 2 cm apart (from center to center) over the muscles in accordance with the SENIAM recommendations [30]. Additionally, electrodes were fixed to the skin with adhesive tape respecting the direction of the muscle fibers. Electrode locations were marked to ensure the consistency in electrode placement between the two days (MVC assessment in the laboratory and track session).

sEMG raw signals were recorded at a sampling frequency of 1000 Hz and amplified a gain of 1000 using an analog differential amplifier and a common mode rejection ratio of 110 dB. A Butterworth bandpass filter of 8–500 Hz (−3dB) was used (RF-Lambda Europe GmbH, Rüsselsheim, Germany). To compute the sEMG amplitude, we used the quadratic mean (root mean square—RMS; µV) at an interval of 0.05 s. The resulting 20 RMS values per second were computed and averaged for the entire duration of each curve (which ranged from 5 to 8 s). sEMG spectra were calculated after fast Fourier transformation with frame width at 1024 and a shift method of 30% of the frame width and selecting the “flat-topped” windowing function. We determined the spectral distribution using the Median Frequency (MF, Hz). The sEMG analysis was performed off-line using the MegaWin Software 2.4. For normalization purposes, both amplitude and frequency spectrum values were expressed as a percentage of sEMG activity recorded during the MVC basal condition.

### 2.5. Experimental Design and Procedures

Before the “on-track” set-up, the rider performed a specific isometric maximal voluntary contraction (MVC) for each muscle. The activity during MVC was used as a reference for further normalizations of the muscle sEMG recordings on the motorcycle. The classical method of measuring the sEMG activity when acting as an agonist, prevailed for the normalization procedure of the eight monitored muscles [31]. All MVCs were performed with the rider in seated position and with flexed elbow (from 120° to 90°, depending on the test). The maximal brake-pulling action above a simulated motorcycle setup was used for the FS [4,24]. ED and CR muscles were assessed using the dorsal flexion of the hand from a flat surface oriented in prolongation of the forearm in prone position. MVC of the BB and TR muscles was carried out with the flexed elbow (90°) positioned next to the body trunk and the forearm in neutral rotation. Isometric push-up action in prone position with the shoulder abducted at 45° and elbow flexed at 90° was used for the PM. DA was assessed with the shoulder and elbow flexed at 90°, with the forearm in neutral rotation during the upwards pushing action. Finally, for DP the rider horizontally abducted his shoulder at 90° during the backwards pulling action.

The track session lasted the whole day. The participant performed a total of seven rounds of 30 min each, intercalated by resting periods of 30 min each. Three alternated rounds were used for the sEMG survey (R1, R2, and R3). The other rounds were performed with the rider free from the sEMG survey. Prior to these three rounds, the pilot did a warm-up round (“sighting laps”), to verify the proper recording of the sEMG system and to decide the settings of the motorcycle. For more detailed timetable information, see Table 1. During each round, the first and last lap (exiting the pit box and flag to pit box, respectively) were systematically discarded, selecting 10 “clean” laps for sEMG analysis. As a result, a total of 30 laps were analyzed during the training session.

### 2.6. Statistical Analysis

For the analysis of the muscle activity in a non-fatigued state, we selected the first three “clean” laps of the first round (R1). One-way Anova was used to compare muscles RMS in the overall six curves. In the second phase of analysis, the same approach was used to compare the RMS signal in the six curves during these three laps. Shapiro–Wilk test confirmed the normal distribution of the data (*p* > 0.15).

When analyzing the fatigue effect throughout the training session every round and for each of the six curves chosen for analysis, sEMG changes (*y*-axis) were assessed over the different laps (*x*-axis). The slopes of both the normalized RMS and MF values were calculated using linear regressions. The significance of the trend for the linear regressions was determined statistically using the F-test. According to the JASA (joint analysis of sEMG spectrum and amplitude) approach, we used the slopes of RMS and MF regression lines, for all curves and for all laps of each round, to create MF–RMS quadrant diagrams. For every diagram, changes in sEMG are indicated on the abscissa for RMS and on the ordinate for MF [18]. According to Luttmann et al. [20] four interpretations are proposed: (1) when dots were located in the upper right quadrant, a state of force increase was considered, (2) if dots were placed in the lower right quadrant, muscle fatigue was assumed, whereas (3) dots in the upper left quadrant indicated recovery and (4) dots observed in the lower left quadrant suggested a force decrement. The level of significance was set at 0.05.

## 3. Results

The visual inspection of the raw sEMG signal recorded lap by lap (Figure 1B) showed high persistence in all the laps (*n* = 30) and confirmed a very strong repeatability of the muscles activation pattern during the whole training session.

### 3.1. sEMG Amplitude (RMS)

**Figure 2:**
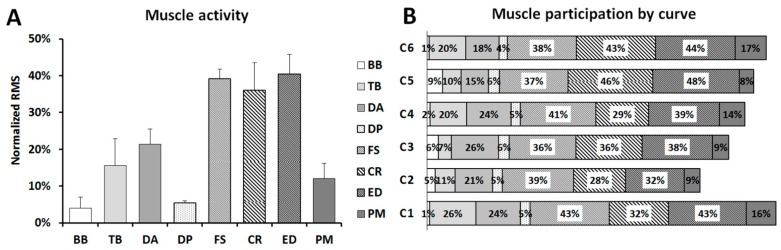
(**A**): Muscle activity of all analyzed curves for the first three laps. (**B**): Muscle participation during the first three laps for each curve. Values are mean and standard deviation.

Analysis of muscle activity before the occurrence of fatigue revealed superior levels of sEMG amplitude in the forearm musculature (ED, FS, and CR) in comparison with the other muscles (*p* < 0.001) (Figure 2A). On the contrary, DP and BB were the less solicited muscles (*p* < 0.001). Finally, it is noteworthy that the RMS values in TB and DA muscles were systematically greater than the activity of DP and BB (*p* ≤ 0.02) (Figure 2A). When comparing the sums of muscle activity associated with different curves (Figure 2B), it seems that C1 and C6 were the most demanding, whereas the lower global RMS values were observed in C2 and C3. Once again, the greatest levels of muscle activity were registered in the forearm muscles, independently of the curve (left or right side) (Figure 2B).

**Figure 3:**
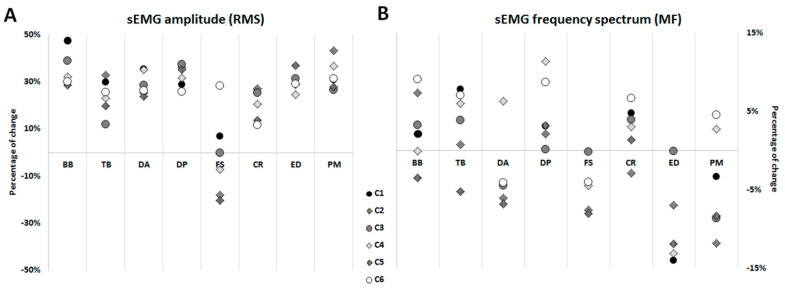
Averaged percentage of change in muscle activity between the first and the last lap of the three rounds, for each curve. (**A**): sEMG amplitude (RMS); (**B**): sEMG frequency(MF). Muscles are: biceps brachii (BB), triceps brachii (TB), anterior and posterior part of the deltoid (DA and DP respectively), flexor digitorum superficialis (FS), extensor carpi radialis (CR), extensor digitorum communis (ED), and pectoralis major (PM).

Once the sEMG amplitudes in a non-fatigued state were described (Figure 2), it was justified to investigate how the occurrence of fatigue could change the activity interplay among these muscles. The averaged percentages of changes in RMS between the first and the last lap of the three rounds for every curve are shown in Figure 3A. The results of linear regressions between RMS and the number of laps, for each curve and round, are shown in Table S1 (Supplementary Materials). The slopes were different from zero (*p* ≤ 0.05) for the majority of the right curves, whereas in the left curves, the statistical significance prevailed from the second round onward. Considering all the curves without exception, more than 89% of the slopes of the graphs representing RMS values were positive in all muscles, except in the FS (67% of the total number of slopes). On the other hand, focusing exclusively on the right curves (C1, C4, and C6), we can observe that all the recordings for this side are characterized by a positive slope for the BB, TB, DA, DP, ED, and PM muscles. The same consistent behavior was observed for the muscles TB, DA, DP, ED, and PM in the left curves (Table S1, Supplementary Materials).

### 3.2. sEMG Frequency Spectrum (MF)

Figure 3B shows the percentage of change in spectral values quantified by the median frequency of all muscle groups investigated along the laps and for each curve. When simple linear regressions were carried out, the data obtained showed a spectral shift to the left in more than half of the significant regressions for DA, FS, ED, and PM muscles, whereas mainly positive slopes were observed for BB, TB, DP, and CR (Table S2, Supplementary Materials). Focusing on the right curves (C1, C4, and C6), we must highlight the strength of the results in the ED muscle, which showed a significant spectral shift to the left (*p* < 0.05) in the majority of occasions, while the frequency spectrum of the TB, DP and CR muscles mostly shift significantly to the right. Nevertheless, the forearm muscles (FS, CR, and ED) systematically showed a significant MF decrement during the last round (R3), with relatively high r^2^ values (0.61–0.82). Considering the left curves (C2, C3 and C5), significant MF increments were observed mainly in C2 and C3 of the second round, whereas MF decrements prevailed in the third round (Table S2, Supplementary Materials).

### 3.3. JASA Method

**Figure 4:**
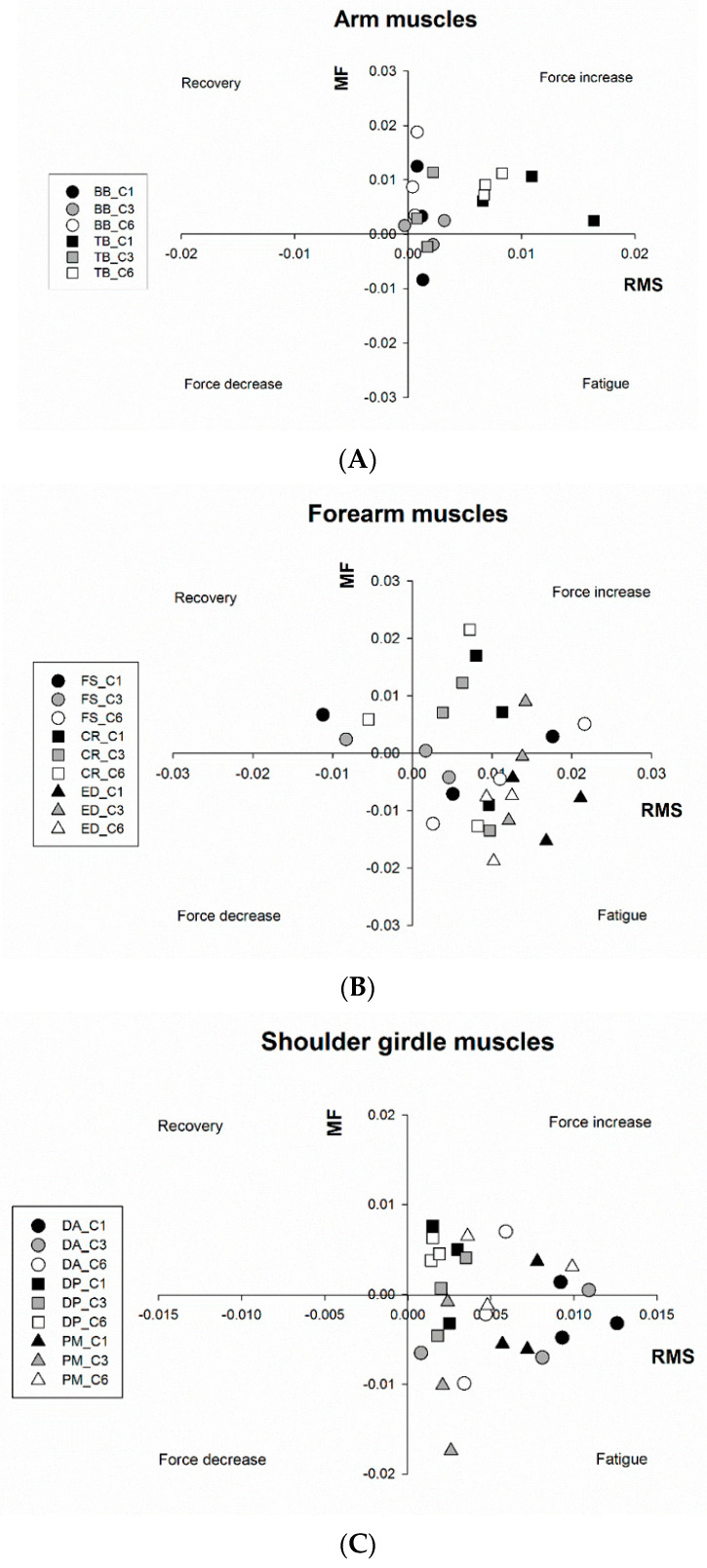
Application of the joint analysis of the sEMG spectrum (ordinate axis) and amplitude (abscissa axis) to the most demanding curves (C1, C3, and C6) with results separated per body segments. (**A**): Arm Muscles (BB: biceps brachii; TB: triceps brachii); (**B**): Forearm muscles (FS: flexor digitorum superficialis; CR: carpi radialis; ED: extensor digitorum communis); (**C**): Shoulder girdle muscles (DA: anterior part of the deltoid; DP: posterior part of the deltoid; PM: pectoralis major). Note: The results of individual linear regressions, for each curve and round, are shown in Supplementary Materials (Tables S1 and S2).

With the Joint Analysis of sEMG Spectrum and Amplitude (JASA) method, all muscles were clearly located in the upper right and lower right quadrants, which are associated with a state of fatigue and force increase, respectively (Figure 4). This kind of representation shows the muscle status grouped by body segments throughout the three rounds, in the most demanding curves (C1, C3, and C6). The fatigue state of the ED muscle, indicated by a simultaneous increase of RMS and decrease of MF, can be observed in 89% of the occasions (Table 2, Figure 4B). The PM and DA muscles also showed a high percentage of occurrence in the “fatigue” quadrant (72% and 61%, respectively), particularly in the left curves (Table 2, Figure 4C). The remaining muscle groups also presented signs of fatigue, although in lesser percentages (from 28% to 44%). On the other hand, arm muscles (BB and TB), DP, and CR revealed a state associated with force increase in most of the sEMG recordings (Figure 4A), mainly in the right curves. This behavior was also observed in the remaining muscles, above all in the right curves. Signs of recovery were found in the CR and FS muscle. The latter showed a great number of braking actions for which the muscular force is not constant, as is clearly demonstrated in Table 2. In summary, while BB, TB, DP, and CR presented a force increase in more than half of sEMG readings, a fatigue state was observed in more than half of the occasions in DA, PM, and in particular ED.

## 4. Discussion

The main objective was to provide, for the first time, sEMG data measured during track motorcycling to define the muscle activity pattern and its changes with fatigue. The results indicated a reliable sEMG pattern for all muscles, with some of them exhibiting signs of fatigue, whereas others showing a progressive increase in the force developed by these muscles across laps and rounds. The main finding of the present study was that in a non-fatigued state, ED, FS, and CR had a substantially higher overall activity than the rest of the analyzed muscles. Additionally, apart from the forearm muscles, TB and DA must be taken into account when riding a motorcycle in an on-track situation. Because of these observations, it is not surprising that these muscles got into a state of fatigue at the end of the track session.

The results confirmed the long-lasting maintenance of an overall considerable activity of the forearm muscles in different layouts of the racetrack throughout the training session. This observation partially explains why a great number of motorcycle riders suffer from the chronic exertional compartment syndrome [28,29].

According to De Luca [16], a stationarity of the signal is needed to ensure that any electrode movement affects the amplitude of the motor unit action potentials (MUAPs) and to guarantee stability in the motor unit activation pattern. During motorcycle riding, it is impossible to maintain the same contraction levels because the rider moves almost constantly during (1) the braking, (2) side-to-side transitions to lean the motorcycle in the curves, and (3) accelerations during the way out of these curves. From the methodological perspective, it is a great advantage that the majority of the movements are performed repeatedly in the same way to move from one position to another and in the same specific sectors of the circuit, lap by lap. When cornering, the rider maintains the position to be as accurate as possible and tries to not make any sudden movements. On that basis, we analyzed the sEMG signals obtained during a real piloting situation both with single electrical indices and using the JASA method proposed by Luttmann et al. [20]. For the JASA method, each action that represents the same activity and/or performed with the same body posture is codified. Following the same rationale [18], we selected sections that involved the same body posture and the same activity. Hence, sEMG signals were consistent and the muscle contractions corresponding to each sector of the track were easily detectable (Figure 1B).

The data obtained for the arm and the shoulder girdle muscles showed that almost all the RMS slopes were significantly and positively associated with the lap number. If we considered only the sEMG amplitude as a fatigue indicator, our interpretation could be that all muscles were fatigued during the entire session. However, the results of the JASA analysis of the overall curves (Table 2) indicated that PM and DA muscles were in a fatigue state most of the time. Focusing on those curves with small radii and preceded by higher velocities (C1, C3, and C6), the fact that PM and DA appear in the fatigue quadrant, especially in the R3, indicates the importance of these muscles for the support of the postero-anterior inertia generated during intense braking [27] as well as for control of the initiation of the “shimmy phenomenon” [32]. Therefore, we suggest that cornering after a straight line where the preceding velocity is higher than 200 km/h implies a very high involvement of the PM and DA muscles, which leads to fatigue. On the contrary, both the TB and DP muscles showed larger force increase behavior than BB (Table 2), although in the hardest curves, the latter showed a predominant state of force increase (Figure 4A). Thus, it is evident that when cornering, these muscles play an important role, probably in withstanding the motorcycle weight and/or in the typical counter-steering maneuver used to modulate the tilt of the motorcycle when getting toward the apex of the curve. Knowing that C2, C3, and C5 were left-sided curves and that the sEMG monitoring was performed only in the right upper limb, it is not surprising that the TB (pushing action during the counter-steering maneuver) was more solicitated in the right-sided curves (C1, C4, and C6) (Figure 2B).

It is also important to consider the movement chosen to test MVC in baseline condition for normalization purposes, before interpreting changes of muscle activity during a global fatiguing task [33]. In the present investigation, it is understandable that the BB, DP, and TB activity recorded during the basal MVC were much higher than during piloting the motorcycle. This kind of normalization procedure may facilitate that these three muscles get toward a state of “force increase” during a high percentage of occasions.

Despite showing state variations, the forearm musculature became fatigued in R3, regardless of the curve analyzed, although the ED muscle seemed to suffer more physical loading during riding than the FS and CR. With few exceptions, the ED muscle in particular was the one which experienced the most fatigue, as demonstrated by the RMS and MF slopes and JASA analysis. This finding supports the results obtained by Torrado et al. [5] who assessed fatigue in the ED muscle after an intermittent fatigue protocol designed for motorcycle riders. These authors suggested, using a motorcycle simulator and protocol duration longer than 30 min, the presence of peripheral fatigue in the ED muscle and changes in cortical excitability, apart from the typical maximal voluntary contraction loss. Nevertheless, this fatigue state was not observed as pronouncedly in the FS and CR muscles, because they changed their position from one quadrant to another (Table 2 and Figure 4B). Such state alternations occurred in these two muscles especially in R2. It seems that professional riders are more habituated to coactivating the aforementioned muscles to improve precision and sensitivity during braking [4,34,35,36]. This behavior was observed in R2, normally considered as the best round from the performance point of view. This was because the rider became accustomed to the track and begins to increase the pace without yet suffering from the external manifestations of fatigue. In fact, the fastest laps tend to be obtained from the second round onward during training sessions. The alternation observed here, precisely in the more technical curves (4 and 5 could thus be explained by the necessity of maintaining the finetuning and precision of the riding in spite of fatigue [4]. Nevertheless, in R3, the fatigue state was clearly present, in agreement with a previous study that confirmed that fatigue was not manifested from the very beginning of a 24 h endurance race [2]. In this study, the normalized MVC was maintained with respect to the basal value during the first two relays, but afterwards it started to decline because of fatigue.

To our knowledge, there has not been a report on such a level of applied analysis in this sport. This study provides the first scientific and direct assessment of what happens when a pilot rides a motorcycle. However, the recording of the gas handle path and of the pressure exerted on the brake lever by a telemetric system of the kind habitually used by the teams of the MotoGP World Championship could upgrade the present study. Hence, this study is a first step towards the assessment of the fatigue during a race situation.

The limitations of this study come obviously from the reduced sample size. Future investigation should address this topic with a greater number of riders and verify if muscle activity patterns, as well as changes of these patterns with the occurrence fatigue, can be generalized or not. On the other hand, the limited number of available sEMG channels (*n* = 8) did not allow us to monitor the left upper limb at the same time. This limitation could certainly cause the underestimation of the muscle activity when cornering the left-sided curves and prevents from comparisons between both limb sides. Another limitation comes from the fact that we used only one racetrack. It could be interesting to monitor the same riders in different racetracks and investigate if the sEMG signal is useful to distinguish different layouts. Finally, it must be mentioned that contrarily to the sEMG amplitude, we must be cautious when interpreting physiological mechanisms derived from changes in the sEMG frequency spectrum. This is because they are not directly related with differences in recruitment and motor unit firing rate of the target muscles [12].

## 5. Conclusions

This study provides descriptive information about muscle behavior during a motorcycle ride on a racetrack. It seems that pushing-like muscles, such as TB and DA, have sufficient relevance as to be seriously considered during physical conditioning specifically oriented to motorcycle racing. Among the always highly demanded forearm muscles, ED was more demanded and fatigued than FS and CR. Whereas PM and DA were fatigued especially in the last round, TB and DP showed a state of force increase. This state was particularly predominant in the BB muscle in the sharpest curves. This study is a step forward towards knowing the fatigue behavior of muscles involved when riding a motorcycle in real racetrack conditions and should help coaches to design specific strength training programs focused on the upper body musculature.

## Figures and Tables

**Table 1:** Timetable of the track session. Riders of two levels (fast and slow) shared alternatively the access to the racetrack. Our rider followed the schedule of the fast (advanced) group (R1–3). Slow riders began their rounds at all half-past hours.

Rounds	Schedule	sEMG Survey
1	from 10:00 a.m. to 10:30 a.m.	warm-up round (“sighting laps”)
2	from 11:00 a.m. to 11:30 a.m.	R1
3	from 12:00 p.m. to 12:30 p.m.	
4	from 13:00 p.m. to 13:30 p.m.	R2
Racetrack closed for lunch time		
5	from 15:00 p.m. to 15:30 p.m.	
6	from 16:00 p.m. to 16:30 p.m.	R3
7	from 17:00 p.m. to 17:30 p.m.	

**Table 2:** Frequency table describing the changes in the Root Mean Square (RMS) and the Median Frequency (MF) of electromyograms derived from the JASA analysis of the whole curves. Each percentage indicates the ratio of occasions when the muscle was assigned to a particular quadrant.

	Number of Curves with:											
	RMS+/MF−			RMS+/MF+−			RMS−/MF+			RMS−/MF−		
	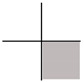			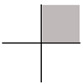			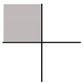			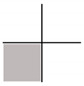		
Muscle	RC	LC	%_TOT_	RC	LC	%_TOT_	RC	LC	%_TOT_	RC	LC	%_TOT_
BB	2	5	39%	7	3	56%	0	1	6%	0	0	0%
TB	1	4	28%	8	5	72%	0	0	0%	0	0	0%
DA	4	7	61%	5	2	39%	0	0	0%	0	0	0%
DP	1	4	28%	8	5	72%	0	0	0%	0	0	0%
FS	4	4	44%	3	1	22%	1	1	11%	1	3	22%
CR	3	4	39%	5	4	50%	1	1	11%	0	0	0%
ED	9	7	89%	0	2	11%	0	0	0%	0	0	0%
PM	5	8	72%	4	1	28%	0	0	0%	0	0	0%
	fatigue			force increase			recovery			force decrease		

RC: right curves; LC: left curves; %_TOT_: the percentage of occasions when the muscle showed such a behavior; BB: biceps brachii; TB: triceps brachii; DA: anterior part of the deltoid; DP: posterior part of the deltoid; FS: flexor digitorum superficialis; CR: extensor carpi radialis; ED: extensor digitorum communis; PM: pectoralis major.

## Data Availability

The data presented in this study is not available to preserve the participant privacy.

## Supplementary Materials

The following are available online at https://www.mdpi.com/article/10.3390/ijerph18157738/s1, Table S1: Linear regression results of sEMG amplitude values (RMS) by rounds for each curve. Table S2: Linear regression results of sEMG frequency spectrum (MF) by rounds for each curve.
