# Supplementary material for: Muscle Fatigue When Riding a Motorcycle: A Case Study
Source: Int J Environ Res Public Health. 2021 Jul 21;18(15):7738. doi: 10.3390/ijerph18157738 (PMC8345341; doi:10.3390/ijerph18157738)
Supplement: Supplementary file 1 [file ijerph-18-07738-s001.zip › ijerph-1285584-supplementary.pdf]

International Journal of
Environmental Research and Public Health

MDPI

Table S1. Linear regression results of sEMG amplitude values (RMS) by rounds for each curve.

			Round 1		Round 2		Round 3				Round 1		Round 2		Round 3	
		Muscle	r^2	Tr	r^2	Tr	r^2	Tr			r^2	Tr	r^2	Tr	r^2	Tr
RIGHT CURVES	CURVE 1	BB	0.59**	↑	0.46*	↑	0.74**	↑	LEFT CURVES	CURVE 2	0.71**	↑	0.58**	↑	0.78**	↑
		TB	0.58*	↑	0.69**	↑	0.90**	↑			0.31	↑	0.49*	↑	0.89**	↑
		DA	0.81**	↑	0.88**	↑	0.86**	↑			0.61**	↑	0.82**	↑	0.48*	↑
		DP	0.90**	↑	0.63**	↑	0.76**	↑			0.77**	↑	0.70**	↑	0.82**	↑
		FS	0.95**	↑	0.44*	↓	0.72**	↑			0.57*	↑	0.72**	↓	0.76**	↑
		CR	0.84**	↑	0.82**	↑	0.64**	↑			0.83**	↑	0.49*	↑	0.68**	↑
		ED	0.64**	↑	0.82**	↑	0.85**	↑			0.78**	↑	0.50*	↑	0.71**	↑
		PM	0.73**	↑	0.55*	↑	0.73**	↑			0.83**	↑	0.68**	↑	0.91**	↑
	CURVE 4	BB	0.43*	↑	0.66**	↑	0.20	↑		CURVE 3	0.01	↓	0.73**	↑	0.79**	↑
		TB	0.58*	↑	0.40**	↑	0.85**	↑			0.17	↑	0.51*	↑	0.79**	↑
		DA	0.50*	↑	0.48*	↑	0.31	↑			0.03	↑	0.83**	↑	0.50*	↑
		DP	0.90**	↑	0.85**	↑	0.84**	↑			0.73**	↑	0.93**	↑	0.38*	↑
		FS	0.26	↑	0.69**	↓	0.93**	↑			0.05	↑	0.58**	↓	0.84**	↑
		CR	0.52*	↑	0.76**	↑	0.69**	↑			0.11	↑	0.74**	↑	0.77**	↑
		ED	0.53*	↑	0.56*	↑	0.83**	↑			0.44*	↑	0.63**	↑	0.79**	↑
		PM	0.21	↑	0.73**	↑	0.79**	↑			0.72**	↑	0.26	↑	0.78**	↑
	CURVE 6	BB	0.72**	↑	0.34	↑	0.89**	↑		CURVE 5	0.21	↑	0.85**	↑	0.73**	↑
		TB	0.43*	↑	0.62**	↑	0.82**	↑			0.03	↑	0.62**	↑	0.87**	↑
		DA	0.56*	↑	0.49*	↑	0.89**	↑			0.25	↑	0.86**	↑	0.78**	↑
		DP	0.58*	↑	0.84**	↑	0.75**	↑			0.68**	↑	0.58*	↑	0.75**	↑
		FS	0.47*	↑	0.84**	↑	0.78**	↑			0.02	↓	0.90**	↓	0.90**	↑
		CR	0.08	↓	0.64**	↑	0.73**	↑			0.03	↓	0.74**	↑	0.81**	↑
		ED	0.26	↑	0.61**	↑	0.52*	↑			0.37	↑	0.73**	↑	0.74**	↑
		PM	0.76**	↑	0.52*	↑	0.63*	↑			0.29	↑	0.56*	↑	0.62*	↑

r^2: coefficient of determination for correlation between RMS and time-course of the laps; Tr: Trend, sign of regression coefficients; ↑ positive regression; ↓ negative regression; * Slopes significantly different from zero ($p < 0.05$); **Slopes significantly different from zero ($p < 0.01$); BB: Biceps Brachii; TB: Triceps Brachii; DA: Anterior part of the Deltoid; DP: Posterior part of the Deltoid; FS: Flexor Digitorum Superficialis; CR: Extensor Carpi Radialis; ED: Extensor Digitorum Communis; PM: Pectoralis Major.

Table S2. Linear regression results of sEMG frequency spectrum (MF) by rounds for each curve.

			Round 1		Round 2		Round 3				Round 1		Round 2		Round 3	
		Muscle	r^2	Tr	r^2	Tr	r^2	Tr			r^2	Tr	r^2	Tr	r^2	Tr
RIGHT CURVES	CURVE 1	BB	0.43*	↓	0.06	↑	0.73**	↑	LEFT CURVES	CURVE 2	0.19	↑	0.21	↓	0.52*	↑
		TB	0.42*	↑	0.54*	↑	0.06	↑			0.18	↑	0.86**	↑	0.38	↓
		DA	0.12	↓	0.02	↑	0.31	↓			0.37	↓	0.32	↑	0.87**	↓
		DP	0.40	↑	0.60**	↑	0.36	↓			0.11	↓	0.43*	↑	0.43	↓
		FS	0.12	↑	0.47*	↑	0.66**	↓			0.51*	↓	0.42*	↓	0.70**	↓
		CR	0.61**	↑	0.84**	↑	0.77**	↓			0.47*	↓	0.84**	↑	0.80**	↓
		ED	0.85**	↓	0.45*	↓	0.64**	↓			0.47**	↓	0.59**	↑	0.60*	↓
		PM	0.06	↑	0.41*	↓	0.28	↓			0.47**	↓	0.29	↓	0.65**	↓
	CURVE 4	BB	0.02	↑	0.67**	↓	0.68**	↑		CURVE 3	0.02	↑	0.05	↑	0.01	↓
		TB	0.54*	↑	0.61**	↑	0.66**	↓			0.07	↑	0.51*	↑	0.06	↓
		DA	0.01	↑	0.20	↑	0.32	↑			0.68**	↓	0.01	↑	0.71**	↓
		DP	0.63**	↑	0.44*	↑	0.00	↑			0.01	↑	0.41*	↑	0.52*	↓
		FS	0.21	↑	0.46*	↓	0.82**	↓			0.00	↑	0.12	↑	0.20	↓
		CR	0.35	↑	0.84**	↑	0.75**	↓			0.55*	↑	0.74**	↑	0.81**	↓

	ED	0.39	↓	0.02	↓	0.61*	↓		0.01	↓	0.60**	↑	0.87**	↓
	PM	0.00	↓	0.10	↓	0.68**	↑		0.53*	↓	0.00	↓	0.72**	↓
CURVE 6	BB	0.13	↑	0.42*	↑	0.71**	↑	CURVE 5	0.00	↓	0.62**	↓	0.51*	↓
	TB	0.64**	↑	0.73**	↑	0.48*	↑		0.58*	↑	0.42*	↓	0.68**	↓
	DA	0.05	↓	0.66**	↓	0.71**	↑		0.37	↓	0.49*	↓	0.33	↓
	DP	0.10	↑	0.42*	↑	0.62*	↑		0.14	↑	0.17	↑	0.37	↓
	FS	0.62**	↓	0.49*	↑	0.78**	↓		0.44*	↓	0.45*	↓	0.88**	↓
	CR	0.45*	↑	0.86**	↑	0.70**	↓		0.56*	↑	0.85**	↑	0.93**	↓
	ED	0.90**	↓	0.69**	↓	0.82**	↓		0.24	↓	0.22	↓	0.86**	↓
	PM	0.10	↑	0.56*	↑	0.02	↓		0.06	↑	0.68**	↓	0.28	↓

r^2: coefficient of determination for correlation between MF and time-course of the laps; Tr: Trend, sign of regression coefficients; ↑ positive regression; ↓ negative regression; * Slopes significantly different from zero ($p < 0.05$); **Slopes significantly different from zero ($p < 0.01$); BB: Biceps Brachii; TB: Triceps Brachii; DA: Anterior part of the Deltoid; DP: Posterior part of the Deltoid; FS: Flexor Digitorum Superficialis; CR: Extensor Carpi Radialis; ED: Extensor Digitorum Communis; PM: Pectoralis Major.
